# Apicomplexan co-infections impair with phagocytic activity in avian macrophages

**DOI:** 10.1007/s00436-020-06900-3

**Published:** 2020-10-08

**Authors:** Runhui Zhang, Wanpeng Zheng, Arwid Daugschies, Berit Bangoura

**Affiliations:** 1 Institute of Parasitology, Centre for Infectious Diseases, Leipzig University, Leipzig, Germany; 2 Albrecht-Daniel-Thaer-Institute, Leipzig, Germany; 3 Department of Veterinary Sciences, University of Wyoming, Laramie, WY USA

**Keywords:** *Toxoplasma gondii*, *Eimeria tenella*, Co-infection, Chicken macrophages

## Abstract

Mixed infections of *Toxoplasma gondii* and *Eimeria tenella* are likely to occur frequently due to the high prevalence of both pathogens in free-ranging chickens. In this study, we investigated the co-occurrence of the two parasites in the same immune-competent host cell towards altered patterns of parasite-host interactions. Chicken blood monocyte–derived macrophages were co-infected with *T. gondii* RH tachyzoites and *E. tenella* Houghton sporozoites in vitro for 24 h. Through monitoring the uptake of pH-sensitive pHrodo™ Zymosan BioParticles (“Zymosan”) by macrophages, we created a three-dimensional model and to analyze quantitatively phagocytosis using confocal laser scanning microscopy. Assessments of parasite populations were performed by qPCR at 2, 6, 12, and 24 h post-infection (hpi). At 6 hpi, phagocytosis was inhibited in the *E. tenella*–infected cultures while no inhibition of phagocytosis was observed due to *T. gondii*. Phagocytosis activity revealed more complex interactions during co-infection. At 12 and 24 hpi, phagocytosis response to “Zymosan” was distinctly weaker in co-infected cells than in all other groups except for cells mono-infected with high doses of *E. tenella* at 24 hpi. By qPCR, significantly reduced numbers of both intracellular parasites were recorded (10-fold) in all infected groups at 2 hpi. At 12 hpi, the *T. gondii* population reached lowest values but dramatically increased by 24 hpi. Our data confirm that macrophage phagocytosis is involved in the control of invasion by apicomplexan parasites in chicken which particularly applies to *E. tenella* infection and it was able to be altered by the co-existing parasites.

**Electronic supplementary material:**

The online version of this article (10.1007/s00436-020-06900-3) contains supplementary material, which is available to authorized users.

## Introduction

*Toxoplasma* (*T.*) *gondii* and *Eimeria* (*E.*) *tenella* are two ubiquitous intracellular parasites in poultry and are both representatives of parasitic coccidia. *T. gondii* infection is often asymptomatic and reported in almost all the warm-blood animals (Harker et al. 2015). Chicken are considered as resistant host with high seroprevalence of *T. gondii* worldwide (Dubey 2010). In contrast, *E. tenella* is one of the most pathogenic protozoan parasites in chicken and may cause severe enteric diseases and lethality. Even sub-clinical enteric coccidiosis reduces economic productivity in chickens (Dalloul and Lillehoj 2006).

Chicken macrophages play an important role as part of the first barrier of the innate immune response against *T. gondii* and *E. tenella* (Hériveau et al. 2000; Unno et al. 2008; Vervelde et al. 1995; Zhou et al. 2013). Macrophages mainly contribute to the clearance and destruction of both intracellular and extracellular pathogens through phagocytosis (Dalloul et al. 2007). However, *T. gondii* can survive by forming a specialized parasitophorous vacuole (PV) in phagocytes such as macrophages (Sibley 1995). There is evidence that protozoan within human or murine macrophages block phagosome-endosome fusion by *Leishmania* or phagosome acidification by *Toxoplasma* (Desjardins and Descoteaux 1997; Goren 1977). Additionally, *T. gondii* within the PV block lipopolysaccharide (LPS)–triggered IL-12 and TNF-α during macrophage phagocytosis (Butcher and Denkers 2002). Regarding *Eimeria*, a previous study into *E. bovis* infection in bovine calves indicates significant macrophage infiltration of the intestinal mucosa (Taubert et al. 2009). In chickens, it was shown that the engulfment of sporozoites by macrophages occurred few hours after infection and sporozoites were more often located within or next to macrophages in previously naïve than in *E. tenella* immune chickens (Challey and Burns 1959; Cox 2001; Vervelde et al. 1996). Chicken macrophages isolated from peritoneal exudates were shown to engulf *E. tenella* sporozoites at 2–3 h post-infection (hpi) (Long and Rose 1976). Sporozoite-bearing macrophages have been reported to possibly transport *Eimeria* spp. sporozoites to other host cells (Challey and Burns 1959; Trout and Lillehoi 1993; van Doorninck and Becker 1957).

Several published studies investigated interaction of cells involved in innate immunity and *T. gondii* or *Eimeria* spp. during mono-infection, few have referred to mutual host-parasite or parasite-parasite interaction with these two coccidian in co-infection models (Hiob et al. 2017). In fact, mixed infections appear to occur frequently in chickens as prevalence of *Eimeria* spp. and seroprevalence of *T. gondii* and. is high, especially in free-ranging chickens (Al-Gawad et al. 2012; Deyab and Hassanein 2005; Lehmann et al. 2006). A recent case reported in Scotland showed co-infection of *T. gondii* and *Eimeria stiedae* in a wild rabbit (Mason et al. 2015). Field studies on co-infections in chickens are currently lacking; however, an experimental in vivo study points at putative interaction of *T. gondii* and *E. tenella* during co-infection (Hiob et al. 2017).

In our recent in vitro study into co-infection of chicken macrophages with *T. gondii* and *E. tenella*, destruction of parasites mainly occurred before 24 hpi (Zhang et al. 2018). *E. tenella* displayed a tendency to increase replication during co-infection with *T. gondii* at 72 hpi in chicken primary macrophage cultures. Besides, at 2 hpi, more intracellular sporozoites of *E. tenella* than *T. gondii* tachyzoites—if by phagocytosis or invasion—were detected (Zhang et al. 2018). The current study aims at enhancing our understanding in host-pathogen and pathogen-pathogen interaction in co-infected chicken primary macrophage cultures. By generating a 3D model, we studied the capacity of macrophage phagocytosis in co-infected and mono-infected cultures. In addition, the invasion and reproduction of both *E. tenella* and *T. gondii* which are likely related to phagocytosis were investigated in the early phase of in vitro infection.

## Methods

### Chicken primary macrophage isolation and culture

Chicken peripheral blood mononuclear cells (PBMC) were isolated from heparinized whole blood of adult female chickens (6-week old), based on the established protocols (Malkwitz et al. 2013) kindly provided by Dr. Braukmann, Friedrich-Loeffler-Institute Jena, Germany. The animal experiments related to the blood sampling were approved by the responsible authorities (Landesdirektion Sachsen, Germany, trial registration number V13/10). Two-milliliter blood was mixed gently with 2-mL phosphate-buffered saline (PBS) containing 1 mg/mL gentamicin (Life technologies, Darmstadt, Germany). Two-milliliter Biocoll® separating solution (density 1077 g/ml; Biochrom AG, Berlin, Germany) was used to separate the PBMCs by centrifugation at 250×*g* for 45 min. Afterwards, the isolated PBMCs were washed with 5-mL PBS once, followed by centrifugation at 350×*g* for 30 min. The resulting pellet was washed and centrifuged (350×*g*, 20 min) once with 5-mL pre-warmed, 41 °C, RPMI-1640 medium (Sigma, Taufkirchen, Germany). Subsequently, 5 × 10^6^ PBMC/well were resuspended in RPMI with 5% chicken serum and 5% fetal bovine serum, penicillin (100 U/mL, PAA), streptomycin (0.1 mg/mL, PAA), and amphotericin B (0.0025 mg/mL, PAA), and incubated in 24-well plates for 96 h (41 °C, 5% CO_2_). The macrophages were purified by rinsing off non-adherent cells twice in PBS at 2 h, 24 h, and 96 h after seeding PBMC cultures for infection.

### Parasites

Genetically modified *T. gondii* RH-GFP tachyzoites (type I strain, kindly provided by Professor Dominique Soldati-Favre, University of Geneva Medical School, Switzerland) were harvested from infected human foreskin fibroblast (HFF) cultures. *E. tenella* Houghton-YFP strain (kindly provided by Professor Xun Suo, China Agricultural University, China) sporozoites were gained following an established protocol (Thabet et al. 2017) with slight improvement. Briefly, sporocysts were collected by mechanical destruction of the oocyst wall with 0.5-mm glass beads (BioSpec Products, Bartlesville, OK, USA). Sporocysts were incubated in 0.25% trypsin (w/v) (Carl Roth, Karlsruhe, Germany) and 4% sodium taurocholic acid (w/v) (Sigma-Aldrich, Taufkirchen, Germany) at 41 °C for 90 min for excystation. Then, sterile pluriStrainer® 5 μm (pluriSelect Life science, Leipzig, Germany) was used to purify excysted sporozoites with 1% glucose in PBS at pH 7.4 (follow buffer).

### Infection

Cell cultures were divided into six groups. Infection groups were performed with a multiplicity of infection (MOI) of certain parasites per cell (Table 1, total infection doses based on the mean population of adherent macrophages/well): Group NC consisted of uninfected negative control PBMC cultures that were seeded 96 h before the start of the experiment. Infection groups were conducted as follows: Groups TH (MOI of 4 *T. gondii* tachyzoites), TL (MOI of 2 *T. gondii* tachyzoites), EH (MOI of 4 *E. tenella* sporozoites), EL (MOI of 2 *E. tenella* sporozoites), and CI (MOI of 2 *T. gondii* tachyzoites and 2 *E. tenella* sporozoites, simultaneous infection). In total, 10 cell cultures from 2 birds were applied repetitively in this study as follows: 6 cultures (3 cultures per bird) for phagocytosis assay and 4 cultures (2 cultures per bird) for qPCR analysis. Groups were observed over a period of 24 h.

**Table 1:** Infection groups

Infection	Group TH	Group TL	Group CI	Group EH	Group EL	Group NC
*Toxoplasma gondii* tachyzoites	5 × 10^5^	2.5 × 10^5^	2.5 × 10^5^	-	-	-
*Eimeria tenella* sporozoites	-	-	2.5 × 10^5^	5 × 10^5^	2.5 × 10^5^	-

### Phagocytosis assay

Twenty-four-well plates for cell imaging (Bottom thickness: 170 μm, Cellvis, CA, USA) were used to assess macrophage phagocytosis according to the manufacturer’s protocol. Briefly, 500 μg/mL of pHrodo™ Red Zymosan BioParticles (“Zymosan”, Life Technologies, USA) were vortexed and resuspended homogeneously in RPMI (pH = 7.4). Cultures were rinsed off in PBS three times to remove extracellular parasites before activation. Each well was supplemented with dispersed “Zymosan” 200 μL/well at 2, 6, 12, and 24 hpi, and incubated at 37 °C without CO_2_ supplementation for further 2 h after induction of activation. Infections were repeated 6 times per time point for each culture. The reaction was washed 3 times with PBS. Then, ice-cold PBS was added to stop the reaction for cell imaging. NucBlue™ Live ReadyProbes™ (Life Technoglogies, USA) were used to stain nuclei. In order to test for basic function of phagocytosis by cells treated according to the described conditions, cultures of group NC (*n* = 6 per observation period) were exposed to activation at 2, 6, 12, and 24 hpi (infection time point for other infection groups). Additionally, uninfected control cultures (*n* = 3) were stimulated for 1 h by LPS (1 μg/ml) application 96 h after seeding in 24-well plates. Afterwards, cells were rinsed with PBS and activated by dispersed “Zymosan” for further 2 h as well. Non-cell controls were kept in parallel. All incubation steps were performed in a dark chamber.

### Confocal laser scanning microscopy

Phagocytosis was determined by cell imaging with × 200 magnification using CLSM (Leica TCS SP8, Wetzlar, Germany). Imaging spots were taken from the central area of six individual wells for each group and observation period. Parasite visualization (× 400 magnifications) was carried out 12 hpi. Stacks were calculated for every 1.5–2 μm and included all attached cells. Defined range of emission for each fluorescence and sequential acquisition (LAS X, Leica, Wetzlar, Germany) was used to avoid overlapping. Imaris® software version 9.3 (Bitplane, Abingdon, UK) was used to generate a 3D model from the stacks and to quantify the number of positive cells with intracellular granules of “Zymosan”. The relative inhibition of phagocytosis in infected cultures following 2 h of stimulation by “Zymosan” was calculated as follows:

$$ \mathrm{Relative}\ \mathrm{in}\mathrm{hibition}=1-\frac{\mathrm{number}\ \mathrm{of}\hbox{'}\hbox{'}\mathrm{Zymosan}\hbox{'}\hbox{'}-\mathrm{positive}\ \mathrm{cells}\ \mathrm{in}\ \mathrm{in}\mathrm{fected}\ \mathrm{group}}{\mathrm{number}\ \mathrm{of}-\mathrm{positive}\ \mathrm{cells}\ \mathrm{in}\ \mathrm{non}-\mathrm{infected}\ \mathrm{control}} $$

### Intracellular parasite replication

Cultures were trypsinized by Biotase® (Biochrom, Berlin, Germany) at 37 °C for 30 min and collected at 2, 6, 12, and 24 hpi after washing three times gently with PBS. DNA was extracted using the QIAamp DNA Mini Kit® (Qiagen, Hilden, Germany) following the manufacturer’s instructions for cell cultures. *T. gondii* DNA standard curve was obtained by gradient 10-fold dilutions of 10^7^ tachyzoites. *T. gondii* replication was analyzed by the 529-bp repeat element in a probe-based qPCR. ITS1 fragment quantification was used to quantify the replication of *E. tenella* by SYBR Green-based PCR (Kawahara et al. 2008).

According to the chosen infection doses, DNA was extracted from 2.5 × 10^5^ and 5 × 10^5^ *E. tenella* sporozoites, respectively, to normalize the initial copy numbers of *E. tenella* (*n* = 4). The relative copy number of *E. tenella* DNA was implemented by measurement of pSCA-17 plasmid standard dilution as described before (Thabet et al. 2015). Quantitative real-time PCR (qPCR) was performed using the CFX Connect Real-Time PCR Detection System (Bio-Rad, Hercules, USA).

For *T. gondii* qPCR, 5 μL of sample DNA eluate was used in a total volume of 25 μL containing 12.5 μL of Master Mix, 3.2 μL of DNase/RNase free water (Gibco™, Life Technologies, USA), 0.9 μL of each 25 μM forward and reverse primer, and 2.5 μL of 2 μM TaqMan probe. The cycling program consisted of 95 °C for 15 min (initial denaturation), followed by 40 cycles of 95 °C for 15 s (denaturation), 60 °C for 1 min (annealing), and 72 °C for 15 s (extension). Two microliters of sample DNA eluate was used for *E. tenella*–specific PCR, in a total volume of 20 μL containing 10 μL of SYBR Green master mix, 7.2 μL of water, and 0.9 μL of 25 μM forward and reverse primer. For *E. tenella* qPCR, the cycling program consisted of heating to 95 °C for 5 min (initial denaturation), followed by 40 cycles at 95 °C for 30 s (denaturation), 55 °C for 20 s (annealing), and 72 °C for 20 s (extension). A subsequent melting curve analysis (95 °C for 1 min, 55 °C for 30 s, 0.5 °C/s) was performed for *E. tenella* qPCR to create the dissociation curve. Data represent the mean of three replicates with an acceptable standard deviation of less than 0.5 for Ct values.

### Data analysis and statistics

The intracellular *T. gondii* tachyzoites were represented as total DNA copy quantities. For *E. tenella*, the DNA copy number/μL was assessed for each observation period. RPN was calculated as mean value (*n* = 3 or 4) with standard deviation (SD) for each group in relation to DNA copy numbers determined for the initial infection dose. Statistical analysis was calculated by SPSS® version 20 (IBM, New York, USA). Differences between groups were determined by the non-parametric Mann-Whitney *U* test and assumed significant at *p* < 0.05.

## Results

### Phagocytosis in uninfected macrophages cultures

**Fig. 1:**
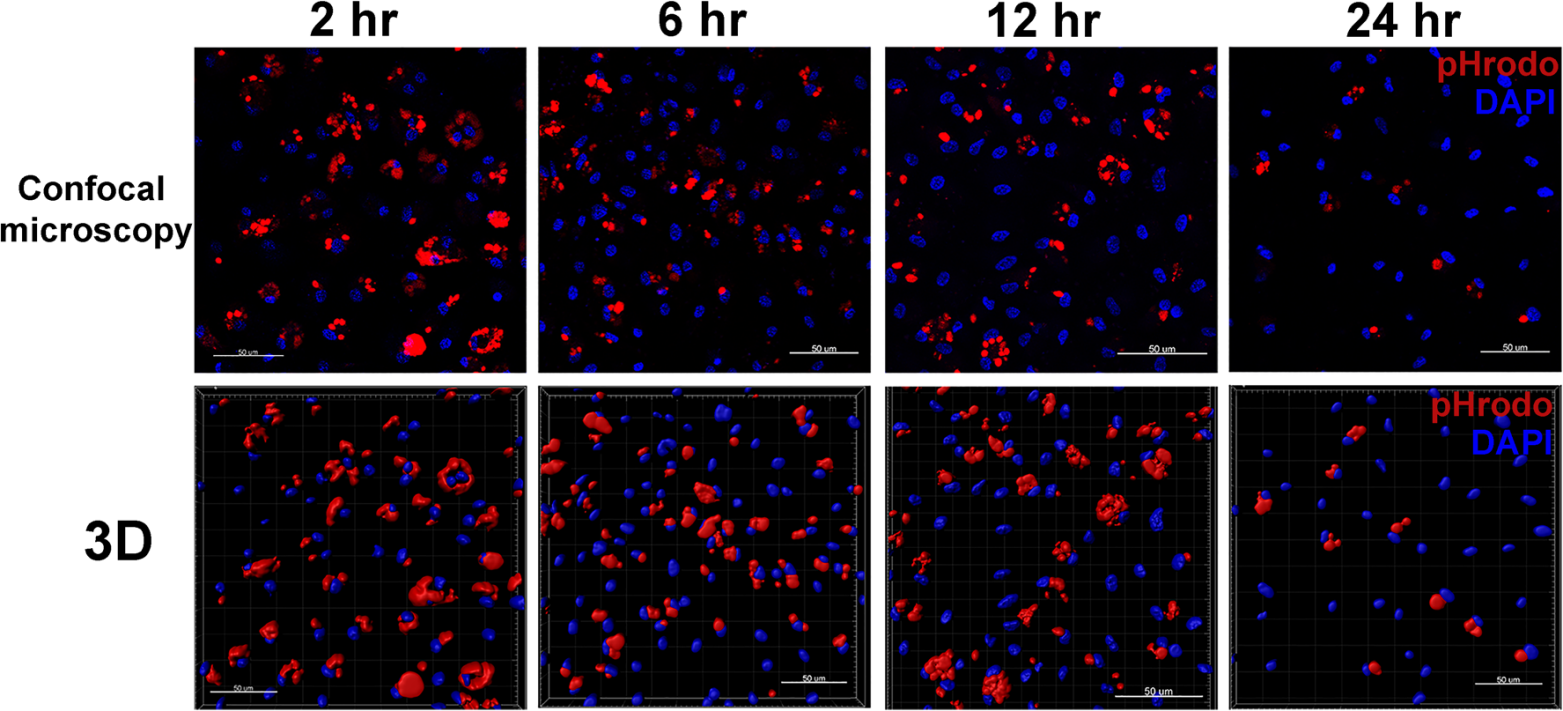
Phagocytosis activity of primary chicken macrophages at 2, 6, 12, and 24 hpi. Phagocytosis was determined after 2-h exposure to “Zymosan” by cell imaging with. *n* = 6 cultures, each 3 cultures per bird. Non-positive staining of “Zymosan” and cell nuclei of macrophages was presented in blank culture (negative control) (data not shown). Staining: DAPI (cell nuclei, NucBlue™ Live ReadyProbes™ blue); pHrodo (“Zymosan” bioparticles, red). Confocal laser Scanning Microscopy (CLSM), × 200 magnifications; 3D model generated by Imaris®

NC phagocytosis was evaluated at time points corresponding to hpi of the infected cultures that were kept in parallel (Fig. 1). Thus, hpi is also used to describe the effects observed in NC although these cells were not infected. During the 2 h of exposure to “Zymosan,” no detectable changes in morphology or signs of apoptosis were found by light microscopy (data not shown). At 2 hpi, more than 70% of DAPI-positive macrophages incorporated “Zymosan” (Table 2). In contrast, LPS exposure at the beginning of the experiment resulted in a clearly lower proportion of activated cells to 53.7 ± 2.8% of DAPI-positive macrophages. In group NC, 57.6 ± 2.0% of cells were phagocytic until 6 hpi, and 56.9 ± 2.5% cells were revealed “Zymosan” positive at 12 hpi. At 24 hpi, phagocytosis percentage decreased to the lowest observed value with 36.6 ± 1.2% (Table 2).

**Table 2:** Phagocytosis of primary chicken macrophages was evaluated using pHrodo™ red Zymosan BioParticles

Further cultivation time point	^a^% of phagocytic macrophages	
	Mean	SD^b^
2 h	71.96	4.83
6 h	57.64	2.02
12 h	56.86	2.49
24 h	36.57	1.08
^c^LPS stimulation at 0 h	53.65	2.83

^a^% of phagocytic macrophages = 100 x (“Zymosan”-positive macrophages/total macrophage positive with nuclei blue); Total macrophages with or without “Zyomsan” were calculated by Imaris® Software, *n* = 6 cultures. Blank well without cell culture was applied as negative control treated with same staining procedures

^b^*SD* standard deviation

^c^*LPS*: lipopolysaccharide, 1 μg/ml, 1 h activation, *n* = 3

### Phagocytosis in infected macrophage cultures

**Fig. 2:**
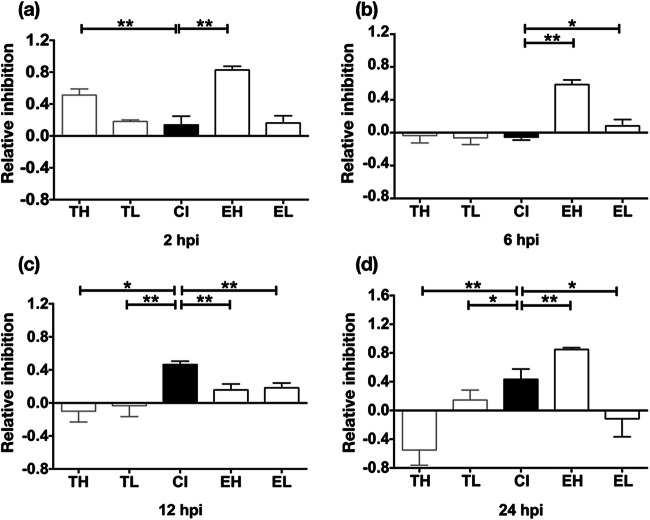
Relative inhibition of “Zymosan” engulfment in chicken macrophages by mono- and co-infection until 24 hpi. Values represent mean values and standard deviation in relation to phagocytosis in group NC (uninfected, *n* = 6, each 3 cultures per bird). Values of relative inhibition equal 0 in group NC. (a) Relative inhibition at 2 hpi. (b) Relative inhibition at 6 hpi. (c) Relative inhibition at 12 hpi. (d) Relative inhibition at 24 hpi. Asterisks (*p < 0.05, **p < 0.005) indicate a significant difference between groups (Mann Whitney *U* test). Denomination of groups: Groups TH (MOI of 4 *T. gondii* tachyzoites), TL (MOI of 2 *T. gondii* tachyzoites), EH (MOI of 4 *E. tenella* sporozoites), EL (MOI of 2 *E. tenella* sporozoites), and CI (MOI of 2 *T. gondii* tachyzoites and 2 *E. tenella* sporozoites, simultaneously infection)

Cell cultures were finally infected up to 24 h as listed in Table 1. At 2 hpi, all infected groups showed initial inhibition of phagocytosis compared with group NC, however, on variable levels (Fig. 2a and Supplementary Material). Inhibition was most pronounced in groups TH and EH, being significantly higher than in group CI (*p* < 0.005). No additive effect was seen in phagocytosis inhibition by co-infection as compared with groups TL and EL that were mono-infected with the same dose of the respective parasite as used for co-infection of group CI.

Over the following investigation period, patterns of inhibition varied.

At 6 hpi, compared with group NC, *E. tenella* infection inhibited macrophage phagocytosis in group EH and, less distinctly, in group EL, whereas *T. gondii* showed no inhibitory effect in groups TH and TL (Fig. 2b). Likewise, no phagocytosis inhibition was observed in group CI. Compared with group CI, both groups EH and EL displayed significantly higher inhibition values (*p* < 0.005 and *p* < 0.05, respectively).

At 12 hpi, Zymosan-induced activation of phagocytosis was similar to group NC in all four mono-infected groups (TH, TL, EH, EL). In contrast, group CI featured a remarkably reduced response to stimulation of phagocytosis compared with group NC, and inhibition values were significantly higher than in all other infection groups (*p* < 0.005 in comparison with TL, EH, and EL and *p* < 0.05 in comparison with TH) (Fig. 2c).

At 24 hpi, group CI remained at a high inhibition level compared with group NC although values were significantly lower than in group EH (*p* < 0.005) (Fig. 2d). In contrast, groups TH and EL did not display reduced phagocytosis, and consequently inhibition values were negative and significantly lower than in group CI (*p* < 0.005 compared with TH and *p* < 0.05 compared with EL). Group TL showed only slight inhibition, although a statistical effect was seen in comparison with group CI (*p* < 0.05). Strikingly, highest phagocytosis inhibition values were observed in group EH, and the respective values were significantly higher than in group CI (*p* < 0.005).

### CLSM

**Fig. 3:**
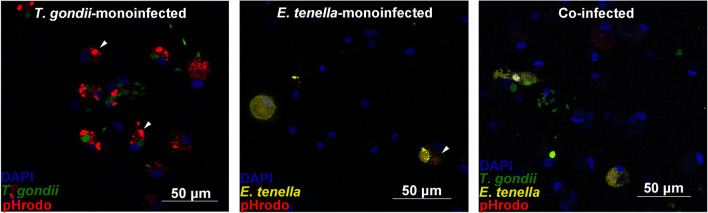
Cell imaging of parasite replication by CLSM at 12 hpi. Typical form of *T. gondii* replication and stage development of *E. tenella* was seen in co-infection at 12 hpi. However, the “Zymosan” was not visualized positively in single co-infected cell. Cell imaging in group NC is presented in Fig. 1. DAPI: cell nuclei stained blue; pHrodo (Zymosan bioparticles) stain red, white arrows; *T. gondii* (tachyzoites, GFP, green); *E. tenella* (sporozoites and meronts, nucleus-stained YFP, yellow)

*E. tenella* meronts and replicating *T. gondii* tachyzoites were visible in all infected macrophage cultures at 12 hpi (Fig. 3). However, fewer intracellular *E. tenella* sporozoites or first generation of *E. tenella* meronts were observed than intracellular *T. gondii* in the respective cultures. “Zymosan”-positive cell was not visualized inside any co-infected cells of group CI.

### Parasite replication

#### *T. gondii*

**Fig. 4:**
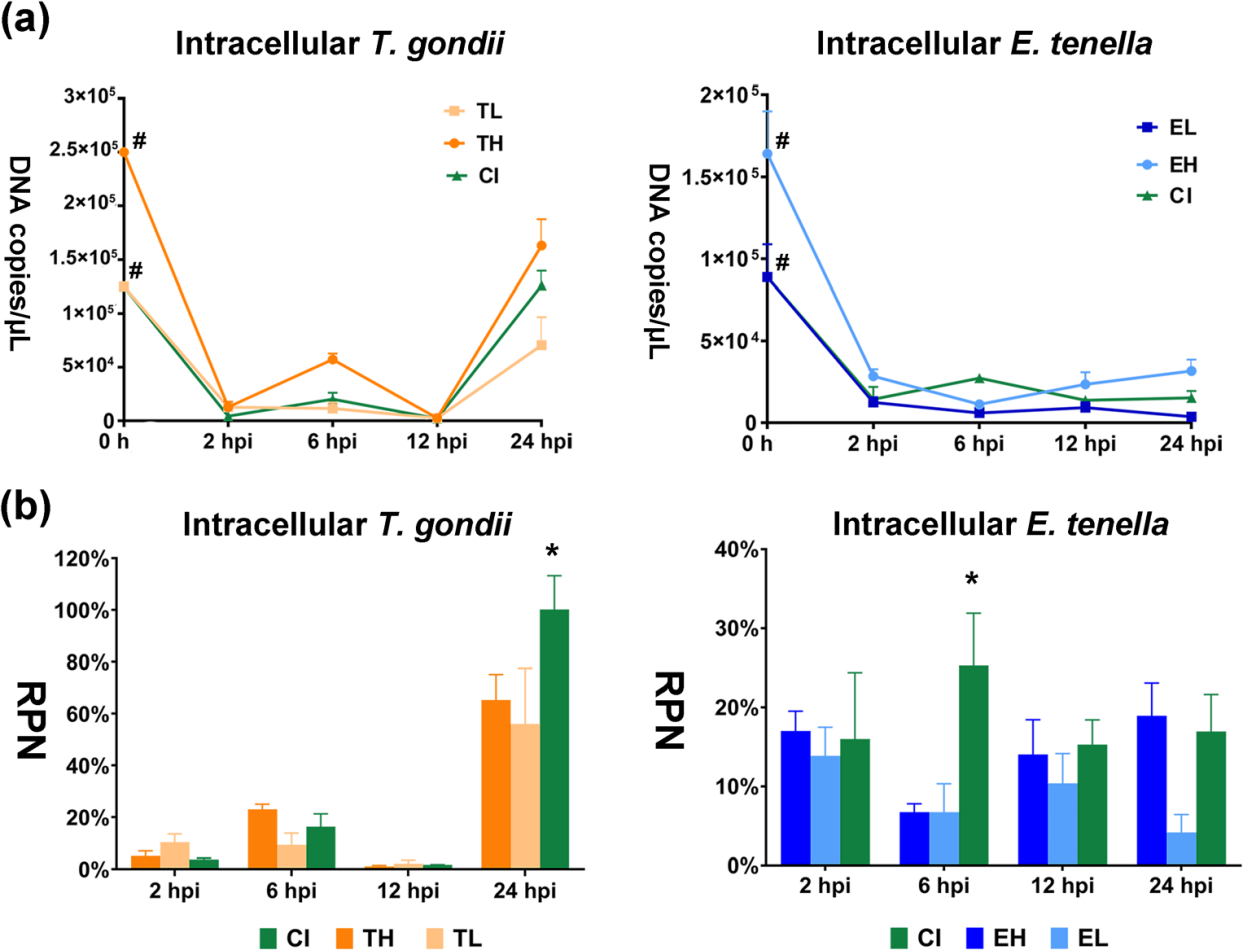
Assessment of parasite reproduction during infection until 24 hpi. (a) Quantities of intracellular parasite estimated by qPCR. *T. gondii* tachyzoites and *E. tenella* (all stages) are represented as DNA copies/μL. Number sign represents DNA quantities of initial infection doses. (b) Relative parasite numbers (RPN) of *T. gondii* and *E. tenella* compared with the initial infection doses. RPN = DNA copies per time point/DNA copies of the initial infection doses. RPN were calculated as mean value of parasite DNA copies with standard deviation (*n* = 3 or 4). *p < 0.05 is considered a statistically significant difference between marked infected groups (based on Mann Whitney *U* test). Denomination of groups: groups TH (MOI of 4 *T. gondii* tachyzoites), TL (MOI of 2 *T. gondii* tachyzoites), EH (MOI of 4 *E. tenella* sporozoites), EL (MOI of 2 *E. tenella* sporozoites), and CI (MOI of 2 *T. gondii* tachyzoites and 2 *E. tenella* sporozoites, simultaneously infection)

Following a rapid initial decrease, low *T. gondii* DNA copy numbers were observed in groups TH, TL, and CI until 12 hpi. However, the quantity of *T. gondii* DNA distinctly increased thereafter by 24 hpi. Interestingly, at the end of the experimental period, group CI reached almost the level of the high-dose mono-infected group (TH) although the infection dose applied was similar to group TL which obviously remained on a lower level of reproduction (Fig. 4a). Relative parasite numbers (RPN) amounted to about 5% in group TH and 10% in group TL at 2 hpi (Fig. 4b). At this time, lowest RPN (3.6 ± 0.1%) were observed in group CI, which were significantly lower as compared with group TL (*p* < 0.05). At 6 hpi, an increase in RPN was observed for group TH (22.9 ± 2.2%), followed by a transient decline at 12 hpi (1.1 ± 0.4%) and finally a steep increase at 24 hpi (65.3 ± 9.8%). In group TL, a similar trend was observed with comparatively low RPN values until 6 hpi (9.3 ± 4.4%) and even a further reduction at 12 hpi (2.1 ± 1.4%), followed by a very distinct increase at 24 hpi (56.4 ± 21.0%).

In group CI, low RPN levels comparable with those recorded for the mono-infected groups TH and TL were observed with lowest values at 12 hpi (1.7 ± 0.1%). However, at 24 hpi, RPN values of group CI (100.1 ± 13.1%) clearly exceeded those of both mono-infected groups (*p* < 0.05). Based on the same initial, *T. gondii* infection dose replication was almost twice as high in the co-infected group as in the mono-infected group TL at the termination of the experiment.

#### *E. tenella*

The quantity of intracellular *E. tenella* stages (sporozoites or first generation meronts) was reduced as compared with the initial dose at 2 hpi and remained low until the end of the experiment irrespective of the applied mono-infection dose (EL, EH) or co-infection (CI; Fig. 4a) without any significant group effects.

Consequently, RPN values were low at 2 hpi with only 13.9 ± 3.6% in group EL and 17.0 ± 2.5% in group EH (Fig. 4b). In both mono-infected groups, RPN levels further decreased until 6 hpi (group EL: 6.7 ± 3.6%; group EH: 6.7 ± 1.1%). Until 12 hpi, RPN moderately increased in these two groups indicating moderately increased reproduction (group EL: 10.4 ± 3.8%; group EH: 14.0 ± 4.4%). This trend continued until 24 hpi in group EH (18.9 ± 4.3%) whereas RPN values had decreased in group EL by then (4.2 ± 2.3%).

Initially, group CI displayed similar RPN (16.0 ± 8.4%) as the mono-infected groups. However, a very distinct peak of RPN values (25.3 ± 6.6%) was noted in group CI at 6 hpi (*p* < 0.05). At 12 hpi and 24 hpi, RPN of CI cultures were rather similar to those of the EH group (15.3 ± 3.1% and 17.0 ± 4.8% at 12 hpi and 24 hpi, respectively) and, at 24 hpi, RPN of group CI, infected with the same *E. tenella* dose as cultures of group EL, were 4-fold higher than in the EL group.

## Discussion

Macrophages are professional phagocytes and highly efficient at internalizing particles (Aderem and Underhill 1999). Flow cytometry and pH-sensitive fluorescent particles have been broadly used to visualize and quantify phagocytosis by macrophage cell lines (Pérez-Flores et al. 2016) or non-adherent phagocytic cells (Gordon et al. 2017; Neaga et al. 2013). However, a recent study (Kapellos et al. 2016) indicated that primary macrophages showed higher sensitivity to external stimulation and could suffer more from stress than other cells during fluorescent staining or running on a flow cytometer. We made similar observations working with chicken primary macrophages (data not shown). Most phagocytosis assays were carried out in mammalian macrophages, so far. However, direct comparison of data from mammalian macrophage cultures to our study is of limited value because the avian immune system features complex differences to the mammalian immune system, i.e., different antigen receptors (Iqbal et al. 2005), number of cytokines families (Kaufman et al. 1999), and delayed NO production of avian macrophages compared with murine macrophages (Malkwitz et al. 2018). So far, only two recent reports were found on the investigation of phagocytosis by using the same pH-sensitive fluorescent bioparticles in a chicken macrophage cell line (HD11 cells) (Garrido et al. 2018; Lee et al. 2018).

In our experiments, we firstly investigated the capacity of cultured chicken primary macrophages to engulf “Zymosan” at four observation periods up to 24 hpi. Macrophage showed great adaptation without observed apoptosis after applying “Zymosan” following the manufacturer’s instructions. Initial engulfment was efficient with on average 72% of macrophages displaying phagocytosis at 2 hpi. However, macrophage activity decreased to 36.6% until the end of the study period. This finding indicates that primary macrophage phagocytosis is generally declining over time. Compared with a recent study of Garrido et al. (Garrido et al. 2018), showing that about 25% of HD 11 cells retain phagocytic activity induced by pH-sensitive bioparticles after 16-h mock treatment, our observed phagocytosis percentages are in a good range over the whole chosen study period.

The cell density of murine bone marrow–derived macrophages (BMDMs) and the duration of activation played a crucial role in uptake of fluorescent particles (Kapellos et al. 2016). Therefore, for longer-term phagocytosis investigations in primary avian macrophages, optimization of cultivation and assay conditions may be crucial to improve phagocytosis capacity over a longer period of time. In our study, LPS polarization led to a reduction of the uptake of “Zymosan” by about 20%. This is in line with the findings in murine BMDMs, where stated that cytokine expression changes by LPS stimulation may reduce their ability to engulf fluorescent particles (Kapellos et al. 2016). Altogether, the model described here appears to be a suitable model to perform investigations into *T. gondii* and *E. tenella* infections including co-infection in chicken macrophages.

Pathogen-pathogen interaction is more and more recognized as an important factor in pathogenesis and disease outcome. For parasites, in vivo studies into enhanced interactions of pathogens co-existing in a host and evidence of competition were shown (Clark et al. 2016; Hiob et al. 2017; Onaga et al. 1983). Regarding coccidia, clinical disease caused by *E. maxima* is ameliorated by concurrent *E. praecox* infections in chickens (Jenkins et al. 2008). *Eimeria* replication in terms of oocyst excretion numbers does not seem to be influenced by co-infections with other *Eimeria* species in most in vivo studies (Cornelissen et al. 2009). However, a study into experimental *E. tenella* infections showed significantly lower oocyst excretion in a group co-infected with *T. gondii* than in an *Eimeria* mono-infected group (Hiob et al. 2017).

In contrast, our previous long-term in vitro study revealed that both *T. gondii* and *E. tenella* were multiplying more effectively in co-infected cultures than in mono-infected cultures over a period of 72 hpi (Zhang et al. 2018). Looking into the related impact on macrophage function as a crucial aspect of the innate immune response, we have identified phagocytosis, which is the major activity of chicken macrophages during pathogen exposure, to be severely impacted by *T. gondii* and *E. tenella* co-infections.

With regard to the mode of invasion, it has been demonstrated that a proportion of *T. gondii* may invade host cells actively while avoiding an efficient phagocytic response through formation of a parasitophorous vacuole (Butcher and Denkers 2002; Morisaki et al. 1995). For *E. bovis*, active invasion is negligible in bovine primary macrophages (Taubert et al. 2009). For both *T. gondii* and *E. tenella,* phagocytosis by the host cell was identified earlier as the major route of pathogen entry (Goren 1977; Rose and Lee 1977).

In our phagocytosis assay, all *T. gondii* or *E. tenella* mono-infected groups showed inhibition of “Zymosan” engulfment at 2 hpi. Inhibition of phagocytosis in the high-dose infected groups (TH, EH) was more obvious than in low-dose infection groups (TL, EL). This is in line with a dose-dependent parasite replication (RPN) as observed for both parasites. Interestingly, co-infected macrophage cultures exhibited a significantly lower level of phagocytosis inhibition than seen in all mono-infection groups. Intracellular parasite quantification demonstrated that more than double *T. gondii* copies were detected in group TL whereas slightly lower *E. tenella* copy numbers were found in group EL compared with group CI, which shows that parasite replication is affected differently for *T. gondii* and *E. tenella* during co-infection. While *E. tenella* displays a higher initial incorporation rate upon co-infection, *T. gondii* appears to feature an impaired invasion rate. According to our recent light microscopical study (Zhang et al. 2018) and live cell imaging results (unpublished yet), most *E. tenella* sporozoites were capable at 2 hpi to enter macrophages while most *T. gondii* tachyzoites “prefer” to adhere to the surface of a macrophage or stay free in the cell culture medium. One previous in vitro study likewise reported the capacity of chicken macrophages to engulf *E. tenella* sporozoites at 2–3 hpi (Long and Rose 1976). Another study showed that a high proportion of live *T. gondii* stayed loosely adherent to host cells without invading and inducing phagocytosis (Morisaki et al. 1995). On the other hand, a previous study showed that most *T. gondii* tachyzoites remained adherent to murine macrophages followed by phagocytic inhibition treatment (Ryning and Remington 1978). We speculate that the initially reduced *T. gondii* RPN in group CI compared with mono-infection groups may be that *T. gondii* endocytosis by macrophages was blocked on the host-cell side by phagocytosis-inhibited macrophages previously entered by *E. tenella*. However, further evidences need to support it by investigating the signal pathway interactions.

Entry of both *T. gondii* and *E. tenella* increased whereas no inhibition was seen on phagocytic capacity of macrophages in co-infection at 6 hpi. Interestingly, co-infection showed distinctly lower phagocytic inhibition but higher RPN of *E. tenella* than *E. tenella* mono-infection group. Meanwhile, phagocytic inhibition by mono-infection of both parasites likely revealed biphasic at 6 and 12 hpi in our study. Specifically, inhibition appeared mostly in the *E. tenella* infection. Due to lack of comparable research, we assumed that significant engulfment of *E. tenella* was associated possibly to the low phagocytosis-inhibited macrophages modulating by *T. gondii* infection.

Combining to our data of quantification and visualization, it appears that a small number of both parasites retained their capability to evade from macrophage phagocytosis and developed successfully while most were eliminated at 12 hpi. Similar results were seen before in mono-infected chicken primary macrophage cultures with the lowest parasite DNA copies of *T. gondii* at the same point (Malkwitz et al. 2017). *T. gondii* and *E. tenella* stages were occasionally identified within the same individual macrophages, which is in line with findings from our previous study (Zhang et al. 2018). *E. tenella* exhibited higher initial infection efficiency than *T. gondii* as inferred from RPN at 12 hpi in relation to the applied infection dose. However, CLSM suggests that most of *E. tenella*-YFP failed to show fluorescence and little intracellular sporozoites or meronts were visualized during *Eimeria* infection. The co-infected cell cultures showed distinctly lower activation by “Zymosan” than mono-infected cultures at 12 hpi. Interestingly, phagocytic activity as measured by “Zymosan” incorporation into macrophages was initially inhibited stronger in both groups EH and TH than in the co-infected group CI which was exposed to both parasites at half the infection dose. This effect reversed at 12 hpi indicating that co-infection leads to more complex effects on a host culture than a mono-infection. Similar to the LPS polarization which was described earlier, co-infection likely triggered immune pathways in the cultures, and stronger so than mono-infections did, which needs to be further discussed.

Relative phagocytosis inhibition values at the end of our study period (24 hpi) were strongly increased in groups EH and CI, while TH cultures displayed significantly higher phagocytosis activity than uninfected cultures. Seemingly, *E. tenella* appears to be mainly responsible for the reduced macrophage phagocytosis that was observed in group CI. Both studied apicomplexan parasites have similar ways of host cell invasion and intracellular replication. Nevertheless, they exhibit different effects on chicken macrophage function related to innate immunity. It may be hypothesized from our data that parasite stage and replication as well as parasite adaption to its host may be involved in the observed infection-related group differences. Follow-up studies including in vivo studies or immune intervention will be needed to investigate the underlying mechanism of phagocytosis inhibition (*E. tenella* and co-infections) or enhancement (*T. gondii* infections towards the end of the study).

## Conclusion

This in vitro infection model of primary avian macrophages can provide an efficient and accurate way to further investigate mechanisms of host-parasite and parasite-parasite interaction during co-infections with pathogens such as monoxenous and heteroxenous coccidia. The current findings contribute to our understanding of macrophage modulation by intracellular parasites and functionality at the early stage of infection with *T. gondii* and/or *E. tenella.*

## Electronic supplementary material

ESM 1 (XLSX 19 kb)

## Data Availability

All data generated or analyzed during this study are included in this published article and its supplementary information files.
